# Learning support strategies for learners with neurodevelopmental disorders: Perspectives of recently qualified teachers

**DOI:** 10.4102/ajod.v9i0.561

**Published:** 2020-02-06

**Authors:** Amarachi J. Yoro, Jean V. Fourie, Martyn van der Merwe

**Affiliations:** 1 Department of Educational Psychology, University of Johannesburg, Johannesburg, South Africa

**Keywords:** inclusive education, neurodevelopmental disorders, recently qualified teachers, mainstream classroom, qualitative research

## Abstract

**Background:**

Inclusive education envisages the improvement of the quality of education for all learners. This further implies that schools must adjust all systems of teaching and learning to accommodate all learners regardless of their diverse needs. The reduction of educational inequalities through inclusive practices is aimed at supporting the accomplishment of academic outcomes for all. Learners presenting with neurodevelopmental disorders (NDDs) place specific requirements on teachers, particularly when they find themselves in mainstream classrooms.

**Objectives:**

This study focused on the learning support strategies used by recently qualified teachers in accommodating learners with NDDs in mainstream classrooms in the Gauteng province of South Africa.

**Method:**

A qualitative approach was used to explore the support strategies used by recently qualified teachers in mainstream classrooms when dealing with learners with NDDs. Purposive sampling was used to select six recently qualified teachers from different mainstream classroom. Data were collected using semi-structured interviews, observations and critical incident reports.

**Results:**

The findings revealed that teachers employ a variety of support strategies such as cooperative learning, peer learning, ability grouping, extensive visual aids and curriculum differentiation in an attempt to support learners. The support provided by the teachers was evident in their performance as learners with NDD were able to learn and understand the lessons irrespective for their barrier to learning.

**Conclusion:**

Contrary to literature findings that teachers do not support learners with diverse needs because of lack of skills, training and knowledge, this study revealed that recently qualified teachers employ a variety of support strategies to support learners with NDDs. However, it appeared that these support strategies were rather general teaching and learning strategies. More support strategies should be applied to help learners with NDD in the mainstream classroom.

## Introduction

Neurodevelopmental disorders (NDDs) are regarded to be multi-dimensional conditions that occur because of abnormal brain development (Mullin et al. 2013). Learners presenting with NDDs show signs of cognition, communication, behaviour and/or motor skills challenges resulting from brain development. Learners with NDDs such as pervasive, cognitive impairments and specific learning disorders present teachers with numerous challenges, such as disruptiveness, excess workload, inability to complete learning outcomes and poor academic performance (Fuchs et al. 2003:158). Teaching a learner diagnosed with NDD is challenging as it requires adequate attention and care until the learner develops to a level of independence (Robinson, Shelton & Malow 2016). These NDDs are even more challenging for a recently qualified teacher as it requires that adequate support is provided to accommodate diverse learning needs in the classroom (Nketsia & Saloviita 2013:14).

Studies have shown that teachers lack the required knowledge and skills to accommodate and support learners with barriers to learning in the classroom (Eloff & Kgwete 2007; Phasha, Mahlo & Maseko 2013). Given the potential challenges newly qualified teachers may experience when teaching learners presenting with NDD, this study explored the understanding and experience of recently qualified teachers as well as the support strategies applied in accommodating and supporting learners with NDDs in mainstream classrooms in their first year of teaching.

## Inclusive education in South Africa

Before the practice of inclusive education in South Africa, Engelbrecht (2006:254) reported that between 1948 and 1994 the only competitor of the policy of education was the state which showed disregard and lack of provision for learners with barriers to learning. Special needs education at that time was provided on a racial basis and equal access to education was inaccessible to all learners. Furthermore, by legislation and policy, the system of education separated children without disabilities from those characterised as having special needs. During this period the South African education practised the traditional medical approach, which labelled learners, tagged and discriminated differently according to race, language, disability, etc. Inclusive education was accepted as a global policy for attending to learners with barriers to learning and diverse learning needs at the Spain Salamanca World Conference on Special Needs (UNESCO 1994). The aim of this conference was not on putting the learners into the school system, rather to change the system of education to ensure equal access to education for all learners. This strategy ensured social justice and equity in order to accommodate the diversity of barriers in the school system (Motitswe 2014:259).

To meet the global movements in inclusive education, the Department of Education (DoE) in South Africa introduced the ‘Education White Paper 6 on Special Needs Education: Building an Inclusive Education and Training system’, which is a policy framework focused on building an inclusive system of education that is focused on the principles of equality for all learners, human rights, equal participation and access to education (DoE 2001).

Implementing inclusive education is determined by the teaching methods and approaches used by teachers to accommodate all learners in the mainstream classroom. The application of inclusive teaching strategies is an important aspect of inclusive education (Florian & Black-Hawkins 2011:815). Inclusive education encourages complete involvement and equality through supporting learners with disabilities from limiting family backgrounds, thereby providing them with an opportunity to participate in the education system (McConkey 2003). Thus, inclusive education is a tool for transformation, a democratic means of understanding values that accept human diversity (Swart & Pettipher 2005). Inclusive schools must have an open arm for all learners who are new to the mainstream school system and must seek to ensure that the environment is receptive to all regardless of their differences in abilities (Maguvhe 2015).

Inclusive education focuses on all subject disciplines and demands that teachers identify, accommodate and support different levels of learning needs of children (Makoelle 2016:60). Inclusive education is about accepting that all children can learn and that they need support to ensure effective learning. This support entails adjusting and restructuring school structures, learning styles and strategies to address and accommodate the different learning needs of learners (DoE 2001). Providing support also includes improving the classroom behaviour of learners, applying a variety of teaching methods, differentiating the curriculum and modifying the classroom environment in order to meet the various needs of learners.

### Teachers’ attitudes towards inclusive education

Makoelle (2016:72) contends that the practice and concept of inclusive education is still a mystery to educators because they struggle to understand what constitutes an inclusive classroom. The state of inclusive pedagogy in South Africa reveals that there is a misconception amongst educators about inclusion and special needs education. This misconception is a result of the dominated paradigm prior to the practice of inclusion in 1994. However, there is a proof that some mainstream schools in the state and independent sector have expressed a desire to implement inclusive education by accommodating learners who ordinarily are meant to be in a special school (Engelbrecht, Oswald & Forlin 2006:122). Many teachers experience stress and are anxious when interacting with learners with additional needs in an inclusive classroom because they lack adequate training to accommodate and support these learners with additional support needs (Engelbrecht et al. 2013:309). Regardless of the commitment of mainstream schools to accommodate learners with additional learning needs, it is also evident that teachers have displayed negative attitude towards implementing inclusivity in the classroom (Nel et al. 2011:76). One major problem experienced is that learners who are meant to be accommodated in mainstream schools often find themselves as a ‘guest’ in the classroom (Walton 2013). This may be because of existing expectations of the inability of the teacher to provide the needed additional support to enable these learners to participate fully in the learning process. The teachers feel unprepared for the practice of inclusive education in the classroom (Hay, Smith & Pauslen 2001).

The major reason behind the existing teachers’ inability to accommodate and support these learners is that they lack adequate knowledge and skills required to accommodate learners with barriers to learning (Mahlo 2017:2). According to Eloff and Kgwete (2007), these teachers admitted having only ordinary diplomas and degrees in education as their pre-service training programmes failed to expose them to the reality of the diverse needs of learners in the classroom. This study further revealed that newly qualified teachers who have been trained in inclusive education exhibit a positive attitude towards learners with NDD and also employ a variety of support strategies in accommodating these learners.

### Learning support in inclusive settings

This study explored teachers’ understanding, experience and various support strategies used in accommodating learners with NDD in mainstream classrooms. Support in the context of this study is regarded as any and all activities that elevate the ability of a school as a system to respond to the diverse learning needs of children. Such support also assumes the availability and readiness of a group of teachers to assist and accommodate learners with barriers to learning (Calitz 2000:22).

‘Learning support’ is a somewhat contested term. For example, in remedial educational contexts, a medical deficit model of diagnosis and categorisation may be followed, which would imply that the ‘learning support’ offered should be the treatment of the deficits that the learners have been diagnosed with. On the other hand, learning support as viewed from the perspective of the socio-ecological model (Dreyer 2013:57) acknowledges the potential learning ability of learners to grow gradually at their own pace to achieve an independent level of learning and personal growth. This growth is supported by using a variety of support strategies, applying different learning styles that suit various learning abilities, by changing systems in the school context and through collaboration with other stakeholders within the school system (Landsberg, Krüger & Nel 2005:145).

In essence, therefore, learners in need of ‘remediation’ or learning support are not separated from the general classroom teaching in inclusive education. Supporting the needs of these and all other learners in the classroom thus becomes the focus of learning support in inclusive settings. Inclusive policies such as the Education White Paper 6 stipulate clearly that learning support for learners with additional needs should be seen as an everyday practice in the classroom and should be provided in such a manner that the barriers to accessing the curriculum and learning opportunities are removed and addressed (DoE 2001). Classroom teachers need to provide support using various teaching strategies that cater for all learners’ active participation during the lesson and through this support learners can interact with the teacher and other learners whilst learning is established (Mittler 2012).

### Neurodevelopmental disorders

Neurodevelopmental disorders are a group of conditions that occur at the beginning of a child’s developmental period before transiting into formal school (Craig et al. 2016). Learners with NDD are challenged with weakness in memory and may have behavioural, motor skills and speech problems. This onset period is known to manifest developmental deficits that produce impairments of personal, social, academic or occupational functioning (Almogbel, Goyal & Sansgiry 2017). Neurodevelopmental disorder is also a genetic or brain condition that causes childhood onset brain dysfunction. Neurodevelopmental disorders manifest in the developmental period and co-occur in individuals with autism spectrum disorder (ASD), attention deficit hyperactive disorder (ADHD), specific learning disorder and intellectual disability.

Neurodevelopmental disorders are prevalent in general classrooms, and the resultant challenges faced by learners presenting with these conditions manifest as behavioural and learning challenges, particularly heightened by ongoing assessment expected in the mainstream (Beckman, Janson & Von Kobyletzki 2016). In the past two decades, there has been a global increase in the number of learners with NDD attending mainstream and public schools (Lanzi et al. 2004:47). The number of learners with autism disorder, for instance, in the USA is considered to be 1in every 68, whilst the numbers in low- and middle-income countries are considered to be much higher as 90% of children with autism disorder are found in these contexts (Franz et al. 2017). Prevalence rates of ADHD, for example, are considered to be 5% for children and adolescents in the South African context (Vogel 2014). Reasons for the increase in NDDs range from genetic associated causes to socio-emotional ones. As indicated earlier, the prevalence appears to be higher in low- to middle-income countries where possibilities of deprivation, genetic and perinatal problems, the occurrence of infectious diseases, immune deficiencies and nutritional factors occur frequently (Vogel 2014). Associated trauma, physical, social and emotional, as well as environmental decay and toxicity are further factors that influence the occurrence of NDDs.

Furthermore, there have been recent advancements in educational policies that promote and advocate for all learners learning together irrespective of their learning barriers. The Education White Paper 6 in South Africa is an example of policies that promote equality in the classroom. Neurodevelopmental disorders found in the mainstream schools are mostly mild or moderate disorders and in rare cases severe as learners with severe or profound NDDs are given special attention in special needs schools (Bishop 2010). Most research on NDDs has focused on only one specific NDD, such as ASD and ADHD specific learning disorders. However, it is evident that all these NDDs are present in the classroom (Romski et al. 2018).

The implementation of the Education White Paper 6 (DoE 2001) has opened the classroom doors to all learners regardless of their barriers or disabilities. Learners with NDD are also expected to be well accommodated as they seem to manifest the most sensitive issues for teachers within inclusive classrooms (Engelbrecht et al. 2003). Teaching mathematics to learners with NDD in Ireland revealed that in a mainstream classroom of 50 learners, 15 were found to have different NDDs, such as ASD, ADHD, dyspraxia and dyscalculia. Autism spectrum disorder and ADHD were described to be invisible in the classroom as they were mild and unidentified (Venkova & McGarraghy 2014). Attention deficit hyperactive disorder is described as a consistent pattern of inattentiveness that hampers development and manifests itself in two or more settings, such as home, school or work. It is known to disrupt the executive functions (focus, memory and action) of a learner’s cognitive processing (APA 2013). In the classroom, learners with ADHD constantly disrupt and distract teaching and learning activities (Sayal et al. 2018). Participants in this study confirmed ADHD to be one of the NDDs evident in the mainstream secondary school classrooms. Webb (2011) claims that learners with mild NDD may never be diagnosed formally and cannot be identified or registered for any kind of supportive interventions.

## Research methodology

Using an interpretative, generic qualitative design (Merriam 2009) newly qualified teachers from six mainstream, secondary schools in Gauteng province of South Africa were purposefully invited to participate in the study. These teachers had recently completed their Postgraduate Certificate in Education and were teaching in inclusive mainstream classrooms, where some of the learners manifested with NDDs such as specific learning disability (SLD) and ADHD. Written informed consent was obtained from the participants and pseudonyms were used to ensure their anonymity. Data were collected over a period of 6 months. The participants in this study consisted of five women and one man. The participants (aged 22–28 years) were representative of each racial group and had been teaching in the mainstream classroom for over 8 months. The schools were all secondary schools. Three schools were located in an urban area serving a high socio-economic class, and the other three schools were in a township area serving low socio-economic class. These schools were purposefully selected because they had learners with NDD in their classrooms with teachers who were recently qualified.

### Data collection

Data were collected using three different methods: semi-structured interviews, observations and critical incident reports. Each of the interviews was carried out individually and lasted for about 45–60 min. The interviews were conducted at a convenient time for the participants to avoid interfering with classroom activities. The interview guide consisted of open-ended questions focusing on three broad themes. The first theme elicited the participants’ knowledge and understanding of NDDs. The nature and kind of NDDs was the focus of the second theme. The third theme described the support strategies used by the teachers in their classrooms to support learners with NDD. The questions asked were direct and flexible, which ensured credibility in interviewing participants (Babbie & Mouton 2007). One of the major advantages of semi-structured interviews is the comprehensiveness, detail and depth of information generated from the participants (Creswell & Poth 2018). The observation was used in collecting data as it is a major technique that offers a first-hand account of the study situation (Merriam 2009). Four out of the six participants were observed, and all observations were done within a 45-min class period. The aim of the observations was to help the researcher explore the kinds of NDD evident in the classroom and to look out for the support strategies used by the participants in accommodating learners with NDD. The observations were guided by a checklist that focused on the teacher’s experience, kinds of NDDs in the classroom, support strategies and other extra notes. The observation was also used as a check on the data collected during the interviews.

The final phase of data collection was done in the absence of the researcher as participants were given a critical incident report document to complete. The purpose of providing this document to the participants was to enable them to record any incident that occurred with learners exhibiting NDD in the absence of the researcher. This document had columns for the teachers to write down the incident that occurred, and how they provided support during the occurrence. There were also provisions for the participants to write down the challenges encountered in the course of the incidence. Critical incident techniques are a step-by-step qualitative approach that offers a practical method of collecting information about human experience and their significance for the people involved (Hughes, Williamson & Lloyd 2007). In the report, participants explained the types of NDDs encountered and the support strategies used in accommodating the learners.

### Data analysis

Six steps of thematic content analysis were applied in analysing the raw data to generate themes (Braun & Clarke 2006). The interviews transcripts, observation field notes and critical incident reports were carefully transcribed, ensuring that data from the audio-recorded interview were correctly typed out. The critical incident reports were transcribed through coding of the reported data and this process helped to easily match up the data with other data sets and themes. Raw data were segmented into meaningful units and coded with a clear description, which amounted to more than a single word, thus not just ‘classifying’ data but awarding and interpreting the meaning as is the convention in the interpretive research paradigm (Denzin & Lincoln 2011). Codes were generated through careful identification of patterns in data that answered the research question and used to establish categories that were refined to make connections between themes and categories to fit logical patterns and possible groupings (Thornberg & Charmaz 2014). Themes were extrapolated by capturing and organising codes that have patterns and are relevant to the research question. The thematic analysis suggests that themes are reported, analysed, interpreted and supported by existing literature. Trustworthiness is a way of ensuring thoroughness in qualitative research without Losing its relevance (Mahlo 2011). The principles of trustworthiness were adhered to throughout the research using informed consent, checking and confirming transcribed data with participants, and clarity of methods used for data collection.

### Ethical considerations

Approval to commence the research was sought from the Ethics Committee at the Faculty of Education, University of Johannesburg, after a scrutinised process. Ethics consideration aimed at protecting the participants’ autonomy and dignity (Babbie 2005). Ethical clearance was obtained from the Ethics Committee at the Faculty of Education, University of Johannesburg (Ethical Clearance Number: 2017-022).

## Findings

### Theme 1: Teachers’ understanding of neurodevelopmental disorders

The responses indicated that teachers understood NDDs well, particularly from a brain dysfunctional point of view. They understood NDD to be a learning problem that occurs during the child’s developmental stage and affects the brain, thereby creating cognitive challenges. Participants also described NDD as brain problems that cause difficulty in reading, writing, learning, exhibiting extreme sluggishness and inability to understand lessons like their peers. This is evident in the following quote from a participant:

‘For me, NDD … is any learning problem that is concerned with the brain. That happened during a child’s development stage and basically like issues that affect the child cognitively. For example, I know a child who developed an NDD because he hit his head on a pool when he was a little child and because of that it affected his learning and all.’ (Participant 6, female, 24 years old)

Neurodevelopmental disorder is a developmental disorder that requires maximum classroom support from the teacher in order to achieve equal learning. Therefore, the participants’ understanding was considered imperative in their application of support strategies to accommodate learners with NDD in the mainstream environment.

### Theme 2: Teachers’ experiences of the different types of neurodevelopmental disorders

This finding presents two sub-themes within the overall theme of teachers’ experiences of the different types of NDDs, which play a role in the support strategies used by the teachers to accommodate learners with NDD. The participants reported ADHD and SLD to be the most prevalent in their classrooms. The participants reported having learners who manifested symptoms of SLD and ADHD, as well as a few learners who were diagnosed with ADHD by an educational psychologist.

#### Teachers’ experience with attention deficit hyperactive disorder learners

Behavioural symptoms such as hyperactivity, excessive noise, unnecessary stubbornness and disruptiveness were part of the observed symptoms reported by the participants. Participant 2 reported the presence of ADHD in her classroom based on the consistent manifestation of ADHD by some learners. She described these learners as experiencing difficulty in concentrating in the classroom, consistently disrupting other learners, moving around and always seeking permission to leave the class without any good reason:

‘For me, in all the classes I have taught I see ADHD as more prevalent. Yes ADHD. It’s from my observation … from the symptoms you can dictate. Like learners moving up and down, always looking for permission to go out, consistently disrupting other learners. You see them very restless … always discussing with classmates … Find it difficult to concentrate and do classwork. With these symptoms yeah … one should dictate that the child has ADHD.’ (Participant 2, female, 25 years old)

Another participant described her experience as having learners manifesting ADHD symptoms and had three learners who were already diagnosed, with one of them being placed on medication. The two participants who reported their experiences both agreed that these symptoms were consistent amongst these set of learners as they lacked the ability to provide minimum concentration to the lessons:

‘I observed them, like when you see a child consistently being up and down. Not seating at a place, being excessively disruptive. Not concentrating at all. So, when you see these signs it [is] easy for you to know. And Yeah, they are about 3 of them with ADHD in my entire classroom and they are diagnosed. One takes medication.’ (Participant 3, female, 23 years old)

#### Teachers’ experience with specific learning disability learners

The major SLD symptoms experienced by participants are difficulties with reading, writing and spelling and difficulties with basic mathematical calculations. The findings were mostly based on the visible manifested symptoms by learners in the classroom as none of these was formally diagnosed by an educational psychologist. According to the participants’ responses, learners in their classrooms struggled to read and write, and this was also accompanied by difficulties in their cognitive functioning. The participants from the township school also acknowledged that the symptoms of inability to read, write and spell correctly were inappropriate considering the age and stage of the learners.

‘This school now is a government township school. The NDD I have really noticed is that of learners who cannot write correctly. It is just too much. Learners here suffer from reading, writing and spelling problems. Yes … I can identify him, but it is not diagnosed but the signs are clear that he has dyslexia …. like he struggles to write correctly, the spellings are way too wrong … for his age, it’s not meant to be so.’ (Participant 1, male, 23 years old)

‘It is basically reading and writing. They mostly do not understand the questions. They find it difficult to read and understand. You need to help them to read. They don’t write nicely at all.’ (Participant 6, female, 24 years old)

During the classroom observation, learners who struggle to read and write were noticed. There were a few learners who had very poor handwriting and struggled to read and this was evident in the English class, as these learners had difficulties in reading the passage given to them as a class activity by their teachers. The researcher also observed extreme spelling errors and very poor writing as part of the difficulties identified in the classroom. In addition, the critical incident reports also confirmed the difficulties learners experienced in writing correctly, especially during class activities and assignments.

Specific learning disability manifests in challenges with learning foundational academic skills like reading, writing and mathematics. It may not necessarily be a result of the absence of teaching or lack of instruction in the classroom, but it affects the basic skills that are vital for learning such as reading single words, reading comprehensive paragraphs, handwriting, spelling, pronunciation and basic mathematical calculations. Problems with these skills may result in difficulties learning in other academic subjects, such as history, science and social studies (Johnson et al. 2010:11).

### Theme 3: Support strategies

This theme highlights the various support strategies used by the participants, which include cooperative learning, visual aids, curriculum differentiation, peer learning, oral assessments and ability grouping.

#### Cooperative learning

Two of the participants reported having been successful in accommodating learners with NDD using cooperative learning. Cooperative learning as a support strategy was used to assist learners with ADHD in the classroom. Participant 6 stated that ‘I use a lot of group work. Like for those learners with ADHD, I make them group leaders so that they can focus at least’.

‘You see with me neh? I don’t put them in alphabetical order, I make them work in groups and I walk round and round my class a lot. I want to see everything; I look at their work to see if they are doing it properly and if they are not getting it well. I hold them during break … like I use all sort of mediums.’ (Participant 5, male, 29 years old)

One teacher explained how learners with ADHD responded well to the leadership role given to them as group leaders; this support strategy helped to increase their attention span and it helped them focus more on the task given. Another participant reported on how cooperative learning was used to maintain discipline in the classroom whilst encouraging equal participation of learners with NDD with other learners. These strategies can be seen as a cooperative learning strategy. Cooperative learning enhances learners’ ability to think creatively and actively engage with each other (Johnson & Johnson 2005:6). In addition, learners with ADHD in the classroom can effectively master the curriculum when tasked to learn in small groups (Murphy, Grey & Honan 2005). Cooperative learning method requires group work and involves learners working with and learning from their peers (Hashim & Kawo 2017:373).

#### Visual aids

Visual aids were used by participants to enhance learners’ understanding of concepts introduced in the classroom. Two of the six participants acknowledged that using visual aids helped learners with NDD to have a better understanding of the subject whilst teaching. Participant 5 reported to have recorded improvement in learners’ classroom participation as well as improved performance during exams.

‘You know I use lots of pictures, so it helps them to understand and identify what we are learning. And you know my kids know how to draw a lot. So, we use drawing a lot to understand stuff while teaching, but so far so good it has not been so bad. Their learning has improved.’ (Participant 4, male, 26 years old)

‘Especially when I use the PowerPoint, the kids love to see images a lot. It makes them understand better. And I get to see it in the way they answer questions during the exams, you can always see that visual aids make them understand things more and more.’ (Participant 5, male, 29 years old)

Visual aids can be used to enhance the understanding of learners, especially when explaining or introducing a new or major concept in the classroom (Mathew & Alidmat 2013:87). In teaching and learning different subjects, the use of visual aids plays a major role in ensuring that learners have a mental picture of the concept taught in the class (Van Staden 2011). Most visual aids are used to clarify different concepts and terms that seem to be too difficult for learners to understand on their own (Ajayi 2008).

#### Curriculum differentiation

Curriculum differentiation was used by the participants to bridge the gaps between the curriculum expectations and the learners’ current content knowledge. One participant reported to have supported learners with NDD through intervention classes which were designed to assist learners who could not read, write and understand basic mathematics during the main lesson. Intervention classes were used to break down teaching into small chunks, using improved strategies such as mind maps, reading and writing exercises, and taking time to explain until the proper understanding of the concept is established. The participants also confirmed that the use of this support strategy had helped in improving the academic performance of learners with NDD in their classrooms.

‘My school has an intervention class, so I have intervention classes for learners who struggle. I try to find better ways of helping them, like taking them through the basics. I try to check where the problem is like some do not know how to use the calculator. I try to teach them how to read and write. Like looking for better pictures, diagrams and making use of mind maps that will make them understand.’ (Participant 5, male, 29 years old)

‘Well … I started this in the first term, particularly the grade 8 learners who dint know how to grasp information. This support I render through my intervention class has helped them a lot even preparing for their exams. These classes have improved their marks.’ (Participant 3, female, 23 years old)

Individualised attention and extra time for learners with NDD were also another support strategy reported by two participants. This was specifically given to learners who struggle to read properly in the mainstream classroom. Allowing extra time, slower pace, individualised attention, simplifying the content and modified seating arrangements for learners with disabilities are also effective support strategies that could be used to differentiate the curriculum:

‘You see with me I am new. I’m still trying. Sometimes I give extra time during classwork, I group all learners who can’t read, and I give them more attention and extra help, so they can at least understand what is going on. I know it is not much but at least that is all I can do for now.’ (Participant 6, female, 24 years old)

These strategies enable learners who struggle to learn at their pace other than being disadvantaged by the curriculum (Dufor 2008).

#### Peer learning

Peer learning was used by the participants to support learners with NDD. The use of peer learning was efficient in helping learners who cannot learn on their own, as most of the times learners feel comfortable asking questions to their peers rather than to the teacher. Peering strong learners with weaker learners was reported as a successful support strategy which encouraged active engagement and participation especially during class activities. As one participant stated:

‘More also, you see this peer learning really works. I never knew all this while. I only realised just last term, especially when I peer the strong and the weak learners together. Like you see them teaching each other and engaging nicely with themselves. It is also another way that these learners have been able to receive support.’ (Participant 4, male, 26 years old)

Cooperative learning may appear to be the same as peer learning because both cases involve learners working together to enhance their learning experience. However, cooperative learning involves learners working in groups to discuss ideas and solve problems together, whilst peer learning is happened when one learner leads or teaches another learner during a given task or class activity. This kind of peer learning is usually used to peer weak learners with stronger learners (So & Brush 2008:320). A better way to explain the difference between these terminologies is that in cooperative learning learners learn together, whilst peer learning helps learners to learn from one another. These two approaches are highly advantageous when used effectively. Both methods develop learners’ oral communication and leadership skills and boost their self-esteem and responsibility. Learners’ attitudes to teamwork are also enhanced, which does not only help their learning process but also future employment and social involvement (Dillenbourg 2002:7).

#### Oral assessments

The oral assessment method was used to support learners with NDD who struggle to read and write age appropriately. Oral assessments assisted to determine learners’ abilities rather than failing them for not being able to read and write. Oral questions are asked based on the subject and the response is used to determine the learner’s level of understanding. One of the six participants supported learners with NDD by providing oral assessments:

‘I also have learners who struggle to read and write correctly, and I then access them orally. And assist them with counting that’s mathematics. So, I bring them to me and I listen to them orally, the moment I see they are able to understand and say something I usually just assess them instead of failing them because of their inability to read and write correctly.’ (Participant 2, female, 25 years old)

Oral assessments are helpful in implementing inclusivity (Huxham, Campbell & Westwood 2012:2). It is more beneficial to learners with SLD as it helps build and retain confidence in themselves despite their weakness in reading and writing (Mccormack-Colbert, Wyn Jones & Ware 2017).

#### Ability grouping

Ability grouping is another kind of support that involves grouping learners according to their academic performance in a subject (Richard & Schmidt 2002). Participant 2 described ability grouping as a helpful support strategy in the mainstream classroom for learners with NDD, as this grouping helps to determine the level of attention needed in each group. Learners who have difficulties in reading, writing and cognitive functioning are kept in a separate group with the aim of receiving personal support, such as extra time, slower pace and individualised attention. The participant explained that for a subject like mathematics where learners struggle to learn, she puts them in groups to know where to pay more attention and provide individual assistance. Participants agreed that grouping learners according to their ability helps them to plan and provide teaching modification and adaptation that suits individual learning needs:

‘What I do is that I group them … like as a teacher you know that this is Group A, B or C … So, from this grouping, you get to know how to handle them during the assessment. This makes it easier in terms of support and during the assessment. Let’s say they are learning maths in their different groups[and] the work is very different. I try to make it easier for those with low cognitive functioning in Group C.’ (Participant 2, female, 25 years old)

‘I group these learners into levels 1, 2 and 3. So these learners in level 1 are the very slow ones like they struggle to read, write slowly, they learn slowly and do not understand anything. I have to take it bit by bit. So, during test or assessments, I take time to explain; I also give them extra time. But the other level does not have any issues with coping, so it’s much easier. It is to help me reach them nicely and where to focus more and pay more attention. You know like I just know that this set of people … I know hey need more of my time and energy.’ (Participant 4, male, 26 years old)

The practice of ability grouping may lead to additional stress for teachers as they are expected to prepare various materials and assessments for the different ability groups (Kim 2012:292). Ability grouping could bring feelings of inferiority and discomfort from the social stigma of being assigned to a lower level. There is a possibility that learners who are placed in lower groups or levels may develop a feeling of ‘learned helplessness’ where they accept the notion of being slow learners and therefore remain that way (Luo & Tsai 2002).

Ability grouping may be helpful in some context especially when teaching any kind of language to learners and it would be useful to continue with this practice (Ireson, Hallam & Hurley 2005:445). These authors argue that changes need to be made in order for ability grouping to be more effective. One better way to bring about this change is to group average to high ability learners together to reduce the degree to which they are grouped. Teachers with poor professional ethics have abused the use of ability grouping to the disadvantage of learners in the lower groups.

## Discussion

This study reports on various support strategies that newly qualified teachers applied in supporting learners with NDDs in mainstream classrooms. What is, however, important to note is that the identification and diagnosis of learners with neurodevelopmental issues in classrooms is not an easy task and requires much more than merely noticing certain symptoms. Learners who are younger than their age may be misdiagnosed as parents and teachers could mistake their immaturity to be ADHD (Elder 2010). Using within-grade standards as a basis for diagnosis may impact negatively older children rather than younger ones. Attention deficit hyperactive disorder may be difficult to identify in older children rather than in young children who may easily exhibit hyperactivity and inattentiveness. Edwards (2009:29) argues that in most cases, both young and old children are over-diagnosed because of their inability to adjust or meet the expectations of the age or grade they find themselves in. In this study, apart from NDDs that were confirmed by medical diagnosis, the researchers agree that teachers may have misdiagnosed the NDDs in the classroom. This is not to say that there is no clear evidence of these symptoms; however, these symptoms could be a result of learning problems resulting from factors such as poverty, social background, poor teaching and illiterate parents. Children with NDDs are often known to be ‘masked’ and therefore they may not be noticed or identified in the classroom (Schwandt & Wuppermann 2016) except when the teacher has a thorough understanding of manifestations of NDDs in the classroom.

The findings reveal that newly qualified teachers have a good understanding of learning support and they were able to accommodate and support learners with NDDs in their mainstream classrooms. This finding contradicts the views of Engelbrecht et al. (2013) and Walton (2013) who reported that teachers are unable to provide support for learners with barriers to learning in mainstream classrooms. The newly qualified teachers in this study were able to provide support, reflecting the effectiveness of their initial teacher training experience. Nel, Nel and Tlale (2015) opined that teachers who understand the nature of learning barriers in their classroom tend to be more positive and provide support for learners with barriers to learning in the classroom.

These teachers understood the different types of NDDs and the importance of including diverse learners in their classrooms. They reported that learners with ADHD and symptoms of SLD were present in their classrooms. Some of the ADHD learners were formally diagnosed and were taking medication. Learners with ADHD manifested with symptoms of inattention, hyperactivity and impulsivity. At the secondary school level, learners manifesting these symptoms experience a major hindrance to effective learning. Many learners displayed symptoms of SLD, such as inability to read, write, spell and do mathematical calculations at age-appropriate grades or levels. These foundational skills are needed to master school subjects such as English, Maths, Economics and Science. These challenges made it difficult for the newly qualified teachers to teach the secondary school curriculum; hence, there was a need to apply support strategies to enable these learners to learn effectively.

The newly qualified teachers were able to provide additional support for these learners by applying a variety of support strategies, such as cooperative learning, ability grouping, individual consultation, close monitoring, oral assessments, differentiating the curriculum, the use of visual aids and peer tutoring. These strategies were reported to have slightly improved the academic performance of learners with NDD as well as ensuring that inclusive education was implemented in the classroom. Teachers were able to identify and apply these support strategies because of their knowledge and understanding of the NDD and supporting diverse learners in the classroom. Because teachers were providing these additional support strategies, pre-service training programmes should focus on these strategies for secondary school teachers. There is still a need for teacher training institutions to deepen the knowledge of pre-service teachers, particularly regarding practical in-class strategies for supporting learners with NDDs. In-depth practical experience and training regarding NDD should be emphasised in pre-service teacher training programmes. The pre-service teacher curriculum should be revisited and restructured to focus more on providing skills, training and knowledge regarding NDDs and current interventions that can be applied in the secondary mainstream classrooms.

## Conclusion

This study explored how newly qualified teachers accommodate and support learners with NDD in mainstream classrooms. Teachers confirmed the existence of ADHD and SLD in secondary classrooms. The outcomes of this study also confirm that newly qualified teachers explore different means of providing support to learners with NDD in their classrooms. Given this contextualised view presented in this article, one may ponder about the knowledge, ability and readiness of newly qualified teachers to aptly identify the symptoms and to adequately address the issues as part of the individual support offered to learners. Becoming aware and proficient as teachers to support learners with NDD in general classrooms cannot be left only to trial and error, collaboration with practising others and parents of learners. Instilling certain abilities and knowledge as part of the armoury teachers enter the profession with falls equally on the training they receive in teacher education programmes. The advent of and subsequent focus on an inclusive pedagogical approach to teaching, where rich learning opportunities are created for all learners, requires teacher education institutions and programmes to ensure that pre-service teachers are enlightened and their knowledge is deepened on how they can effectively provide additional support for learners with NDD. Equipping teachers in this way may have a far-reaching effect on how learners are supported towards basic foundational literacy and numeracy skills in mainstream classrooms.
